# Targeting Oxidative Stress, NLRP3 Inflammasome, and Autophagy by Fraxetin to Combat Doxorubicin-Induced Cardiotoxicity

**DOI:** 10.3390/ph14111188

**Published:** 2021-11-20

**Authors:** Ahmed M. Kabel, Samir A. Salama, Almokhtar A. Adwas, Remon S. Estfanous

**Affiliations:** 1 Department of Pharmacology, Faculty of Medicine, Tanta University, Tanta 31527, Egypt; 2 Division of Biochemistry, Department of Pharmacology, College of Pharmacy, Taif University, P.O. Box 11099, Taif 21944, Saudi Arabia; s.salama@tu.edu.sa; 3 Department of Pharmacology, Faculty of Medicine, Sabratha University, Sabratha P.O. Box 250, Libya; almokhtar.adwas@sabu.edu.ly; 4 Anatomy and Embryology Department, Faculty of Medicine, Tanta University, Tanta 31527, Egypt; remon.estfanous@med.tanta.edu.eg

**Keywords:** fraxetin, doxorubicin, cardiotoxicity, oxidative stress, autophagy, rats

## Abstract

Doxorubicin belongs to the class of anthracycline antibiotics that is widely used in the treatment protocols of a wide range of malignancies. The major deleterious effect of doxorubicin use is the possible occurrence of cardiotoxicity. This study aimed to delineate the possible effects of targeting oxidative stress, NLRP3 inflammasome, and autophagy by fraxetin on doxorubicin-induced cardiac dysfunction in rats. In a model of doxorubicin-induced cardiotoxicity, the effects of different doses of fraxetin were assessed by determination of biochemical, histopathological, immunohistochemical, and electron microscopic changes. Fraxetin, in a dose-dependent manner, was found to have the ability to mitigate the harmful effects of oxidative stress and inflammation on myocardial muscles with significant decrease in NLRP3 inflammasome, augmentation of autophagy, and amelioration of the apoptotic signaling pathways. In addition, fraxetin, in a dose-dependent manner, had the ability to combat the echocardiographic, histopathological, immunohistochemical, and electron microscopic changes induced by doxorubicin in cardiomyocytes. As a result, fraxetin may be put into consideration as a new adjuvant line of therapy on the way to mitigate doxorubicin-induced cardiotoxicity.

## 1. Introduction

Doxorubicin is an anthracycline antibiotic that exerts its effects via suppression of topoisomerase II activity, thereby inhibiting important key steps in DNA replication and repair [1]. Doxorubicin acts to a variable extent on the different phases of the cell cycle but the S-phase seems to be the most sensitive to the cytotoxic effects of doxorubicin [2]. These unique properties might confer efficacious antitumor effects to doxorubicin, making it one of the most widely used anticancer agents in a wide range of malignancies [3]. However, the possible development of overt cardiotoxicity even with the therapeutic doses of doxorubicin (60 to 75 mg/m^2^ body surface area) may stand as an obstacle against the desirable effects of doxorubicin as a potent anticancer agent [4]. Over the last decades, several mechanisms were incriminated by scientists to be responsible for this cardiotoxicity, including increased generation of reactive oxygen species (ROS) with significant detriment of the antioxidant defense mechanisms in cardiac tissues [5]. In addition, the direct effects of doxorubicin on nuclear factor kappa-B (NF-κB) expression with modulation of the balance between the pro-inflammatory and the anti-inflammatory cytokines were proven to be responsible for the toxic effects of doxorubicin on cardiomyocytes [6]. Moreover, downregulation of neuregulin-1, which is considered as a key element in the cell survival pathways in cardiac tissues, by doxorubicin, was proposed as an important contributing mechanism [7].

Recent reports paid attention to the salient role of the interaction between the Nucleotide-Binding Domain-Like Receptor Protein 3 (NLRP3) inflammasome, autophagy and apoptosis in the pathogenesis of doxorubicin-induced myocardial dysfunction [8]. Doxorubicin was proven to enhance activation of the NLRP3 inflammasome, which in turn activates the mammalian target of the Rapamycin (mTOR)/AKT signaling pathway with subsequent inhibition of autophagy in the cardiomyocytes [9]. In addition, the enhanced expression of NLRP3 inflammasome was reported to amplify the apoptotic signals in the cardiomyocytes both directly via increased activity of the proapoptotic proteins and indirectly via modulation of transforming growth factor beta 1 (TGF-β1)/Smad 3 signaling, which consequently mitigates the activity of antiapoptotic proteins [10]. Interestingly, agents that combat NLRP3 inflammasome activity were proven to significantly ameliorate the pathogenic effects of doxorubicin on cardiomyocytes without reducing its anti-cancer activities [8].

Fraxetin is a simple coumarin that is extracted from Cortex Fraxini [11]. The ongoing reports throw light on the detrimental effects of fraxetin on tissue inflammation and edema induced by cytotoxic agents [12]. Moreover, fraxetin was proven to affect the complexes of the electron transport chain and modulate the generation of ATP at the mitochondrial level [13]. In addition, fraxetin serves in plants as a key regulator of iron homeostasis which may explain, at least in part, its potent antioxidant and free radical scavenging activities [14]. Recent findings established a relation between iron homeostasis and regulation of the activity of NLRP3 inflammasome in cardiac tissues. These reports might make the potential effects of fraxetin on doxorubicin-induced myocardial dysfunction a hot topic for ongoing scientific research [15,16]. This work was a trial to delineate the possible effects of targeting oxidative stress, NLRP3 inflammasome, and autophagy by fraxetin on doxorubicin-induced cardiac dysfunction in rats.

## 2. Methods and Materials

### 2.1. Chemicals and Drugs

Doxorubicin hydrochloride was supplied by Bio-Techne Co., Minneapolis, MN 55413, USA (CAS # 25316-40-9). Fraxetin was purchased from Molnova, Ann Arbor, MI 48104, USA (CAS # 574-84-5). All other reagents and chemicals were provided as a kind gift from Sigma Pharmaceutical industry, Quesna, Egypt, and were of analytical grade. Both doxorubicin and fraxetin were dissolved in normal saline.

### 2.2. Animals and Groups

This study was conducted on thirty-two male adult Wistar rats weighing about 160–240 g purchased from the animal house of the Faculty of Science, Tanta University, Egypt. The animals were given two weeks to acclimatize to the surrounding circumstances before starting the experiments. Rats were kept at a constant temperature of 24 ± 3 °C with relative humidity of 57 ± 10%, and were subjected to 12 h light/dark cycles. The protocol of this study was conducted following the Helsinki declaration of animal ethics and was approved by the Research Ethics Committee of Faculty of Medicine, Tanta University, Egypt (Approval code 34402/1). A laboratory technician who was blinded of the identity of the experimental groups randomly divided the animals into four equal groups as follows: (I) The control group was injected intraperitoneally with 0.5 mL normal saline twice weekly for two weeks; (II) the untreated doxorubicin group was injected intraperitoneally with doxorubicin (4 mg/kg) twice weekly for two weeks [17]; (III) the Fraxetin small dose + Doxorubicin group received fraxetin by gastric tube in a dose of 40 mg/kg/day for one week before and continued for two weeks concomitantly with doxorubicin injection [18]; and (IV) the Fraxetin large dose + Doxorubicin group received daily oral fraxetin by gastric tube (80 mg/kg) for one week before and continued for two weeks concomitantly with doxorubicin administration [19].

### 2.3. Assessment of Cardiac Function Tests in Blood Samples

At the end of the experimental period, pentobarbital sodium was used by intraperitoneal injection (50 mg/kg, single dose) [20] to facilitate blood withdrawal from the retro-orbital plexus. Blood was centrifuged at 3000 rpm for 15 min for separation of serum, which was utilized for assessment of lactate dehydrogenase (LDH) using kits supplied by Biomatik, Wilmington, DE 19809, USA (CAT # EKN46642) according to the vendor’s guide. Kits purchased from BioVision, Milpitas, CA 95035, USA, were utilized for quantification of the levels of serum creatine kinase (CK-MB) and serum troponin I (CAT # E4608 and E4737, respectively) using the producer’s instructions. Analysis of the levels of N-terminal pro-B-type natriuretic peptide (NT-pro BNP) in serum samples was carried out using ELISA kits obtained from Novus Biologicals, LLC, Centennial, CO 80112, USA (CAT # NBP2-68139) following the vendor’s guidelines.

### 2.4. Processing and Preparation of Cardiac Tissues

Rats were euthanized and cardiac tissues were extracted. A portion of the extracted tissues was homogenized by a Teflon homogenizer (Thomas Scientific, Swedesboro, NJ 08085, USA) and the homogenate was centrifuged at 3000 rpm for 15 min. The resulting supernatant was utilized for exploration of the levels of the biochemical parameters in the specimens of cardiac tissues. The other portion of the cardiac tissue was processed for further histopathological and electron microscopic examination and immunohistochemical staining.

### 2.5. Evaluation of Oxidative Stress Parameters and Nuclear Factor Erythroid 2-Related Factor 2 (Nrf2) Content in Cardiac Tissues

Cardiac total antioxidant capacity was quantified using ELISA kits purchased from Abcam, Waltham, MA 02453, USA (CAT # ab65329) following the vendor’s instructions. Kits obtained from KyvoBio, Evere, Belgium (CAT #MBS268427-96) were utilized for assay of malondialdehyde (MDA) levels in cardiac tissues. Nrf2 content of the cardiac tissues was quantified using kits supplied by Elabscience, Houston, TX 77079, USA (CAT # E-EL-R1052) following the manufacturer’s instructions.

### 2.6. Determination of Cardiac Tissue Transforming Growth Factor Beta 1 (TGF-β1), Interleukin 10 (IL-10), Interferon Alpha (IFN-α), and Nucleotide-Binding Domain-Like Receptor Family, Pyrin Domain-Containing 3 (NLRP3) Inflammasome

Kits purchased from Abcam, Waltham, USA (CAT # ab119558 and ab214566), were utilized for assessment of tissue TGF-β1 and IL10 levels, respectively. IFN-α levels were quantified using kits supplied by CUSABIO, Houston, TX 77054, USA (CAT #CSB-E08637r). Assay of NLRP3 levels was executed using kits provided by Aviva Systems Biology Co., San Diego, CA 92121, USA (CAT # OKCD04232). Determination of the fore-mentioned parameters was carried out following the vendor’s protocol.

### 2.7. Assay of p38 Mitogen-Activated Protein (p-MAP) Kinase, Phosphoprotein Kinase B (p-AKT), and Phospho-Mammalian Target of Rapamycin (p-mTOR) in Cardiac Tissues

Kits provided by RayBiotech, Peachtree Corners, GA 30092, USA (CAT #CBEL-P38-2-RB and PEL-AKT-S473-1) were employed for assay of p-MAP kinase and p-AKT, respectively, according to the vendor’s instructions. Phospho-mTOR levels were quantified using kits purchased from Boster Biological Technology, Pleasanton, CA 94566, USA (CAT #EKC2466) following the provider’s protocol.

### 2.8. Quantification of Beclin-1, Nerve Growth Factor Beta (NGF-β) and c-Jun NH2-Terminal Kinase (JNK) Activity in Cardiac Tissues

Beclin-1 levels were determined in cardiac tissues using kits supplied by Elabscience Biotechnology, Houston, TX 77079, USA (CAT # E-EL-R1122-ELS). Cardiac tissue NGF-β levels were quantified using kits provided by Biomatik, Wilmington, Delaware 19809, USA (CAT # EKF58099). Kits purchased from Abcam, Waltham, MA 02453, USA (CAT # ab273417) were employed to assay JNK activity in the cardiac tissues. Determination of the fore-mentioned parameters was executed following the vendor’s protocol.

### 2.9. In Vivo Echocardiographic Examination of Rats of the Different Studied Groups

In vivo murine echocardiography was performed to evaluate cardiac functions in awake rats. Briefly, rats were imaged in the M-mode using a linear array ultrasound probe (Sonoscape, Centennial, CO 80112, USA) both at the start and end of the study. These images were utilized to determine dimensions and thickness of the left ventricle and to calculate indices of the cardiac functions. The determined parameters included heart rate, left ventricle end-diastolic diameter (LVEDD), left ventricular end systolic diameter (LVESD), left ventricular fractional shortening (LVFS), left ventricular ejection fraction (LVEF), and myocardial performance index (MPI) [21].

### 2.10. Evaluation of the Histopathological Changes in Cardiac Tissues

Specimens of the cardiac tissues were fixed in 10% formaldehyde solution, then put in paraffin blocks. After that, these specimens were deparaffinized by xylene, hydrated in alcohol, stained with hematoxylin for 10 min, and then counterstained with 1% eosin solution. These sections were examined by using light microscope (Olympus Corporation, Lombard, IL 60148, USA) to assess the histopathological changes.

### 2.11. Immunohistochemical Staining of Cardiac Tissues for Assessment of Caspase-3

The cardiac sections were stained with the primary antibody polyclonal IgG to caspase-3 (Cloud-Clone Corp., Katy, TX, USA, product No. PAA626Ra01) and the slides were examined under light microscope (Olympus Corporation, Lombard, IL 60148, USA). The percentage of the positive immunostaining for caspase-3 was calculated using IHC profiler tool in image J software (1.49v) (National institute of health, USA) and was graded as follows: (+) refers to mild immunoexpression, (++) denotes moderate immunoexpression, and (+++) means marked immunoexpression.

### 2.12. Assessment of the Electron Microscopic Changes of the Cardiac Tissues

After fixation of the cardiac specimens in 2.5–4% glutaraldehyde solution (pH 7.4) for 48 h at 4 °C, these specimens were cut into small particles and washed with distilled water. After that, they were fixed in 1% osmium tetraoxide with 15 mg/mL of potassium ferrocyanide for 1–2 h at 4 °C. The tissue specimens thereafter were cut with an ultramicrotome to sections of 0.5–1 μm thickness and stained with uranyl acetate and lead. After that, these sections were examined and photomicrographs captured using a JEOL, JEM 1010 electron microscope (Jeol Ltd., Tokyo, Japan).

### 2.13. Statistical Analysis of the Obtained Data

The obtained data were analyzed and statistically evaluated using Graph Pad Prism version 7.0. Comparisons between the different studied groups were executed using one way analysis of variance (ANOVA), followed by Tukey’s multiple comparison test. Data were referred to as mean ± standard deviation (SD) and the significance was considered at *p*-value of less than 0.05.

## 3. Results

### 3.1. Fraxetin, in a Dose-Dependent Manner, Combatted the Changes Induced by Doxorubicin in Cardiac Function Tests

Doxorubicin-treated rats exhibited significant increase in serum CK-MB, LDH, troponin Im and NT-pro BNP, relative to the control group. Fraxetin was found to have the ability to elicit significant decrease in these parameters, when compared to rats treated with doxorubicin alone. The improvement in the cardiac function tests was more pronounced in rats that received 80 mg/kg fraxetin, compared to the group that received 40 mg/kg fraxetin (Table 1).

### 3.2. Fraxetin Augmented the Antioxidant Defense Mechanisms and Restored Nrf2 Content of Cardiac Tissues in Doxorubicin-Treated Rats

**Figure 1:**
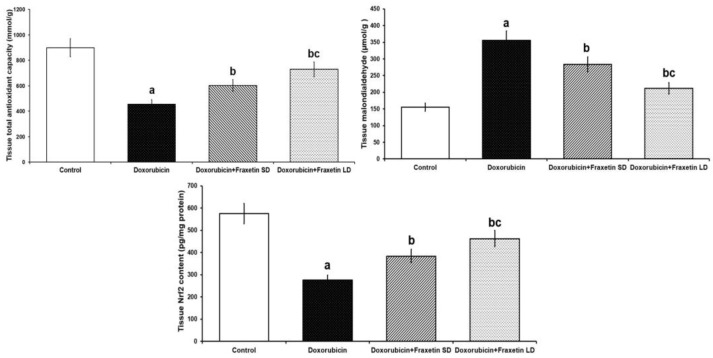
Effect of the different doses of fraxetin on total antioxidant capacity, malondialdehyde, and Nrf2 content of cardiac tissues in doxorubicin-treated rats (Mean ± SD). ^a^ Significant relative to the control group (*p*-value less than 0.05); ^b^ Significant relative to doxorubicin group (*p*-value less than 0.05); ^c^ Significant relative to doxorubicin + fraxetin small dose group (*p*-value less than 0.05).

The group that was injected with doxorubicin alone exhibited significant increase in MDA levels and significant decrease in total antioxidant capacity and Nrf2 content in cardiac tissues, compared to the control group. Administration of fraxetin elicited significant decrease in MDA levels with significant increase in the total antioxidant capacity and Nrf2 content of cardiac tissues relative to rats treated with doxorubicin alone. These changes were more evidenced with 80 mg/kg fraxetin, compared to the group that received 40 mg/kg fraxetin (Figure 1).

### 3.3. Fraxetin Mitigated the Changes Induced by Doxorubicin in Cardiac Tissue TGF-β1, IL-10, IFN-α, and NLRP3 Inflammasome

**Figure 2:**
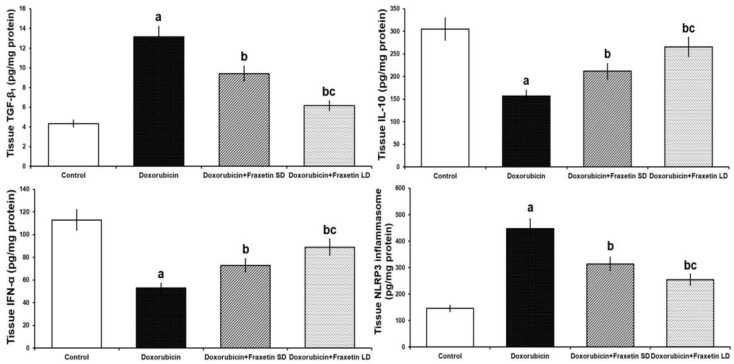
Effect of different doses of fraxetin on cardiac tissue TGF-β1, IL-10, IFN-α, and NLRP3 inflammasome in doxorubicin-treated rats (Mean ± SD). ^a^ Significant relative to the control group (*p*-value less than 0.05); ^b^ Significant relative to doxorubicin group (*p*-value less than 0.05); ^c^ Significant relative to doxorubicin + fraxetin small dose group (*p*-value less than 0.05).

The injected doxorubicin elicited significant increase in cardiac tissue TGF-β1 and NLRP3 inflammasome with significant decrease in IL-10 and IFN-α levels when compared with the control group. Fraxetin, in a dose-dependent manner, had detrimental effects on TGF-β1 and NLRP3 inflammasome levels with significant increase in IL-10 and IFN-α levels resulting in amelioration of the inflammatory processes compared to rats treated with doxorubicin alone (Figure 2).

### 3.4. Fraxetin Augmented the Autophagy-Associated Pathways in the Cardiac Tissues of Doxorubicin-Treated Rats

Animals injected with doxorubicin exhibited significant detriment of autophagy evidenced by significant decline in cardiac tissue p-MAPK and beclin-l with significant inhibition of JNK activity relative to the control group. This was accompanied with significant elevation of cardiac tissue p-AKT and p-mTOR in doxorubicin-treated group compared to the control animals. Treatment with different doses of fraxetin was able to significantly ameliorate these changes with significant increase of p-MAPK, JNK activity and beclin-l levels associated with significant mitigation of p-AKT and p-mTOR levels relative to rats treated with doxorubicin alone but the maximal enhancement of autophagy was encountered with the high dose of fraxetin (80 mg/kg) (Table 2).

### 3.5. Fraxetin Abrogated the Apoptotic Changes in Cardiac Tissues of Doxorubicin-Treated Rats

**Figure 3:**
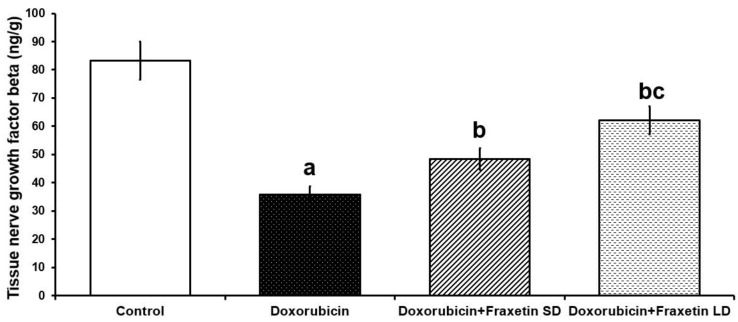
Effect of the different doses of fraxetin on nerve growth factor beta of the cardiac tissues in doxorubicin-treated rats (Mean ± SD). ^a^ Significant relative to the control group (*p*-value less than 0.05); ^b^ Significant relative to doxorubicin group (*p*-value less than 0.05); ^c^ Significant relative to doxorubicin + fraxetin small dose group (*p*-value less than 0.05).

**Figure 4:**
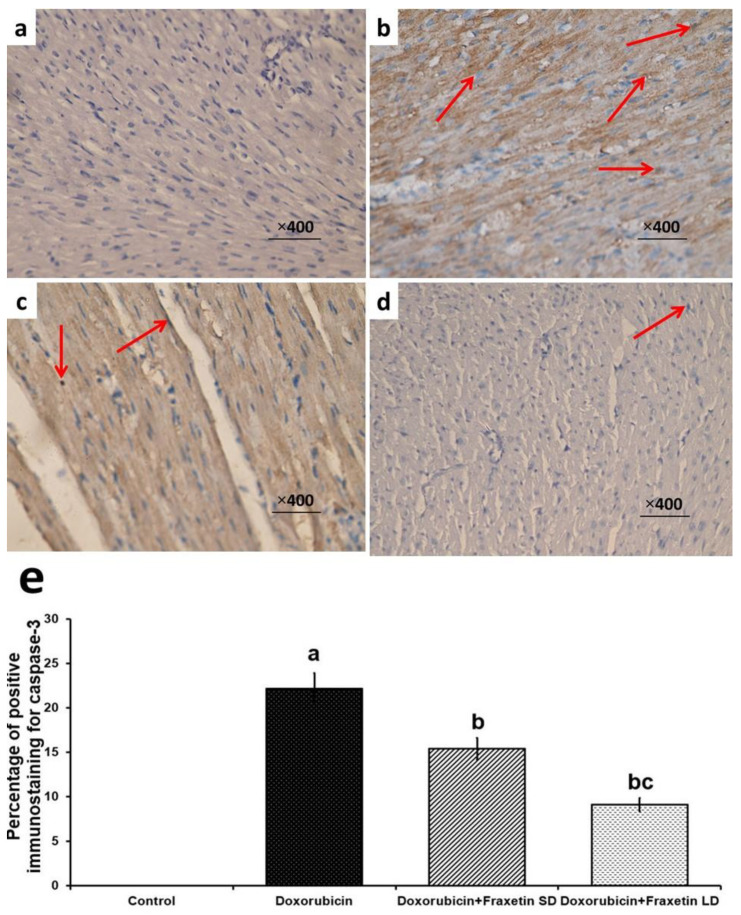
Immunohistochemical staining of caspase-3 in the left ventricle of (**a**) the control group showing negative immunoexpression of caspase-3; (**b**) Doxorubicin-treated group showing strong positive expression of caspase-3 in the form of deep brown discoloration of the cytoplasm of the cardiomyocytes (Arrows); (**c**) Doxorubicin + Fraxetin small dose group showing weak uptake of the stain by the cardiac myocytes, which appeared as mild brown discoloration of their cytoplasm (Arrow); (**d**) Doxorubicin + Fraxetin large dose group showing mild positive immunoexpression of caspase-3 (Caspase-3 × 400); and (**e**) Percentage of positive immunostaining for caspase-3 (^a^ Significant relative to the control group; ^b^ Significant relative to doxorubicin group; ^c^ Significant relative to doxorubicin + fraxetin small dose group).

Apoptosis was significantly enhanced in doxorubicin-treated group, manifested by significant decrease in NGF-β (Figure 3) and significant increase in caspase-3 immunoexpression (Figure 4), compared to the control group. The different doses of fraxetin used in this study had the ability to evade the apoptotic changes with significant increase in NGF-β expression (Figure 3) and significant decline in caspase-3 immunoexpression (Figure 4), compared to rats treated with doxorubicin alone. These changes were significantly encountered in the group treated with 80 mg/kg fraxetin relative to the group that received 40 mg/kg fraxetin (Figure 3 and Figure 4).

### 3.6. Effect of Doxorubicin with or without Fraxetin on Echocardiographic Indices in the Studied Groups

At the baseline of the study, the echocardiographic indices, including heart rate, LVEDD, LVESD, LVFS, LVEF, and MPI were within the normal range for all rats. At the end of the study, the group that was injected with doxorubicin alone exhibited significant increase in LVESD with significant decline in LVEF, LVFS, and MPI, compared to the control group. These changes were reversed with administration of the different doses of fraxetin but maximal improvement was pronounced in rats that received 80 mg/kg fraxetin. No significant differences were noticed regarding heart rate or LVEDD at the end of the study between the different groups (Table 3).

### 3.7. Fraxetin Combatted the Histopathological Changes Induced by Doxorubicin in Cardiac Tissues

**Figure 5:**
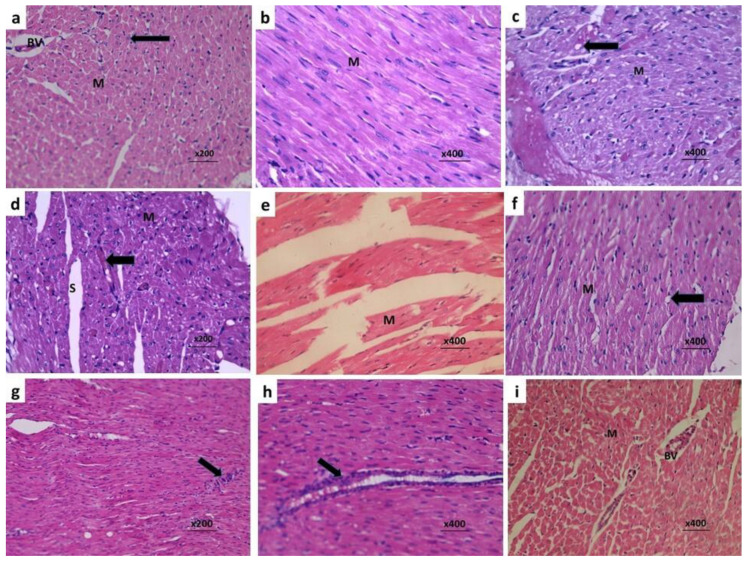
Sections of the cardiac tissues from (**a**) The control group showing transversally cut section of some cardiac muscle fibers (M) with centrally located nuclei (Arrow) and normal-sized blood vessels in between (BV) (H&E × 200); (**b**) The control group showing some cardiac muscle fibers appear longitudinally cut (M) with acidophilic cytoplasm and basophilic central nuclei. The cardiac muscle fibers appear connected and closely adherent (H&E × 400); (**c**) Doxorubicin group showing transversely cut cardiac muscle fibers (M) with marked cytoplasmic vacuolations (Arrow) (H&E × 400); (**d**) Doxorubicin group showing transversely cut cardiac muscle fibers (M) with wide spaces between them (S) and evident interstitial hemorrhage (Arrow) (H&E × 200); (**e**) doxorubicin group showing longitudinally cut cardiac muscle fibers, which appear disorganized into widely separated fragmented bundles (F) (H&E × 400); (**f**) Doxorubicin + fraxetin small dose group showing preserved normal architecture of the cardiac muscle fibers, which appear longitudinally cut (M) with mild cytoplasmic vacuolation (Arrow) (H&E × 400); (**g**) Doxorubicin + fraxetin small dose group showing mild interstitial cellular infiltration (H&E × 200); (**h**) Doxorubicin + fraxetin large dose group showing mild perivascular cellular infiltration (H&E × 400); (**i**) Doxorubicin + fraxetin large dose group showing transversally cut cardiac muscle fibers (M) with centrally located nuclei and normal blood vessel (BV) in between (H&E × 400).

Massive infiltration of cardiac tissues with different types of inflammatory cells with fragmentation of the myocardial fibers were observed in rats treated with doxorubicin alone (Figure 5c–e). Fraxetin administration induced significant reduction in inflammatory cellular infiltration with restoration of the normal architecture of the myocardial fibers (Figure 5f–i). These favorable effects were more pronounced in the group treated with the high dose of fraxetin (80 mg/kg) (Figure 5h,i) relative to rats treated with 40 mg/kg fraxetin (Figure 5f,g).

### 3.8. Fraxetin Abrogated the Electron Microscopic Changes Induced by Doxorubicin in Cardiac Tissues

**Figure 6:**
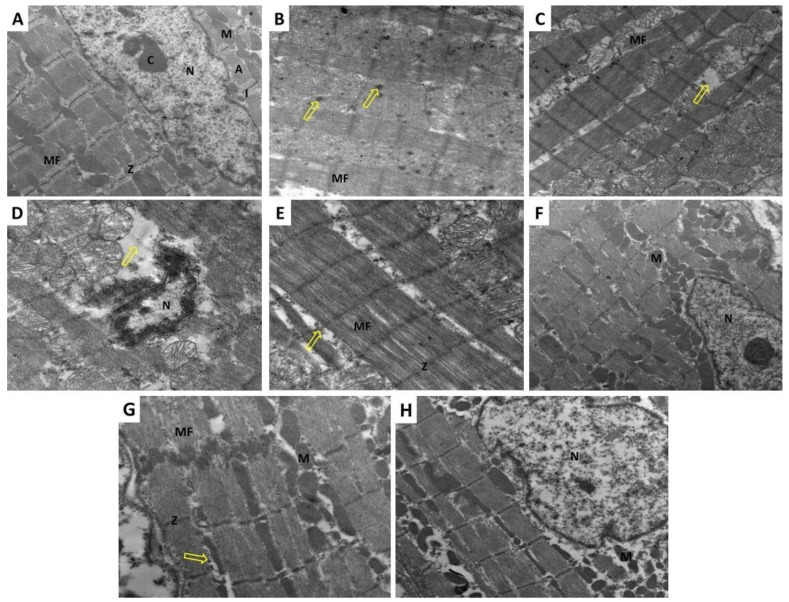
A transmission electron micrograph of a part of the cardiac muscle from the left ventricle of (**A**) The control group showing regular arrangement of the myofibrils (MF) in the sarcomeres between the Z-lines (Z) with arrangement of the mitochondria (M) in rows between them. The dark A-band appears in the middle (A) and the light I-band appears in the periphery (I) of the sarcomere. The nucleus is oval (N) with heterogenous chromatin (C) (Direct mag × 3000); (**B**) The control group showing regular arrangement of the myofibrils (MF). Also, scattered glycogen granules (arrow) appear between the myofibrils and in association with mitochondria are seen (Direct mag × 5000); (**C**) Doxorubicin-treated group showing disruption of the normal architecture of the cardiac muscles. The cardiac myofibrils (MF) are fragmented and widely separated. The mitochondria are of different sizes and depletion of mitochondria in some areas can be seen (arrow) (Direct mag × 3000); (**D**) Doxorubicin-treated group showing shrunken nucleus (N) with irregular outlines and peripheral chromatin condensation. The perinuclear zone shows few mitochondria (arrow) (Direct mag × 5000); (**E**) Doxorubicin + fraxetin small dose group showing apparent restoration of the normal architecture of the cardiac myscles. The myofibrils (MF) are regularly arranged within the sarcomeres between the Z-lines (Z). The mitochondria (M) are normally arranged in between. Some thinning out of the myofibrils and separation are seen (arrow) (Direct mag × 5000); (**F**) Doxorubicin + fraxetin small dose group showing indentation and mild peripheral chromatin condensation of the nucleus (N). Apparently normal mitochondria (M) are seen in the peri nuclear zone (Direct mag × 3000); (**G**) Doxorubicin + fraxetin large dose group showing regular arrangement of the myofibrils (MF) of the cardiac muscles in the sarcomeres between the Z-lines (Z) with arrangement of the mitochondria in rows between them (M). Scattered glycogen granules in association with the mitochondria are seen (arrow) (Direct mag × 5000); (**H**) Doxorubicin + fraxetin large dose group showing an oval nucleus (N), which appears normal in size with regular nuclear envelope and abundant perinuclear mitochondria (M) (Direct mag × 3000).

Administration of doxorubicin-induced marked disruption of the normal cardiac architecture with fragmentation and wide separation of the myofibrils and depletion of the mitochondria (Figure 6C). Also, doxorubicin injection resulted in marked irregularities of the nuclear membrane with shrunken nuclei showing peripheral chromatin condensation (Figure 6D). Fraxetin, in a dose-dependent manner, was able to improve the electron microscopic changes induced by doxorubicin with significant decrease in the irregularities of the nuclear membrane and chromatin condensation (Figure 6E,G) and restoration of the normal architecture of the myofibrils and the mitochondria (Figure 6F,H).

## 4. Discussion

Anthracycline antibiotics represent a large group of drugs that inhibit topoisomerase II enzyme, and thus have been used effectively in the treatment of a large scale of malignancies worldwide [22]. Among the members of this group, doxorubicin represents the most widely used agent with high efficacy against certain types of tumors, including breast, head, and gastric malignancies [23]. The high incidence of myocardial dysfunction that was encountered even with therapeutic doses of doxorubicin attracted the attention of scientists for years; the exact etiology of this undesirable adverse effect remains not fully explored [24]. With marvelous developments in research resources in the last decade, strong evidence has emerged that suggest that doxorubicin affects the expression of certain genes related to the generation of ROS and the pro-inflammatory cytokines with the end result of distortion of the normal architecture and functions of cardiomyocytes [25]. This was in accordance with the data obtained from the current study where rats injected with doxorubicin exhibited significant deterioration in cardiac functions, manifested as significant elevation in serum LDH, CK-MB, troponin I, and NT-pro BNP together with loss of the normal architecture of cardiac tissues and disturbed echocardiographic indices, when compared to the control group.

The pathways that regulate Nrf2 signaling were thought to play a fundamental role in the pathogenesis of doxorubicin-induced cardiotoxicity [26]. Doxorubicin was proven to decrease Nrf2 content in cardiac tissues with subsequent increase in the generation of free radicals and ROS in cardiomyocytes, which subsequently impair myocardial functions [27]. In addition, Cheng et al. [28] postulated that doxorubicin by its detrimental effects on the Nrf2/HO-1 content of the myocardium may significantly decrease the activity of the cardiac antioxidant enzymes with subsequent augmentation of the effects of oxidative stress on cardiac tissues. Moreover, Nrf2 signaling was proven to regulate NF-κB expression, which consequently affects the inflammatory cascade in cardiac tissues [29]. This is in line with the results of the current work, where doxorubicin injection was associated with significant decline in Nrf2 content and total antioxidant capacity associated with significant elevation of MDA content of cardiac tissues, compared to the control group.

In the current study, administration of fraxetin to doxorubicin-treated rats induced a dose-dependent significant increase in Nrf2 content and total antioxidant capacity with significant decline in MDA levels in cardiac tissue, when compared to rats treated with doxorubicin alone. This was in accordance with the findings of Najmanová et al. [30] who threw light on the strong antioxidant properties of fraxetin in various tissues of the body. Kundu et al. [31] attributed these properties to the effect of fraxetin on Nrf2/HO-1 expression, which subsequently affects the antioxidant defense mechanisms. Interestingly, the increase in tissue Nrf2 content induced by fraxetin was concomitantly associated with inhibition of the production of ROS, resulting in abrogation of its harmful effects on the different tissues of the body [32].

Recent studies threw light on the interesting role of NLRP3 inflammasome in the pathophysiology of anthracycline-induced myocardial dysfunction [8]. NLRP3 inflammasome is a multimeric protein complex that is activated by various stimuli, including danger-associated and pathogen-associated molecular patterns [33]. Upon its activation, NLRP3 inflammasome triggers an inflammatory form of cell death, increases the production of TGF-β1, and enhances the release of a wide variety of the proinflammatory cytokines [34]. Wei et al. [35] reported a strong involvement of ROS/NLRP3 inflammasome signaling in doxorubicin-induced cardiac dysfunction. They found that injection of doxorubicin enhances the activity of NLRP3 inflammasome in the myocardium with subsequent hypersecretion of IL-1β and an increase in caspase-1 activity denoting enhancement of the inflammatory events and apoptosis of the cardiomyocytes. This coincided with the data obtained from the current work where doxorubicin induced significant increase in the expression of NLRP3 inflammasome and this was associated with significant elevation of TGF-β1 levels and significant detriment in the expression of IL-10 and IFN-α together with enhancement of apoptosis in the cardiac tissues compared versus the control group.

Coinciding with the results of the present work, Chen et al. [36] reported that administration of coumarins, including fraxetin and isofraxidin, was associated with significant decrease in NLRP3 inflammasome at the gene expression level. Di Stasi [37] attributed this decrease to the ability of fraxetin to inhibit the main initiators of activation of NLRP3 inflammasome including danger-associated and pathogen-associated molecular patterns. In addition, the ability of fraxetin to inhibit NF-κB expression may have a regulatory role on NLRP3 inflammasome production and activity [38]. An interesting finding was that coumarin derivatives, including fraxetin, may enhance the production of IL-10 and IFN-α which are well documented to abrogate the inflammasome-driven augmentation of the inflammatory process [39,40]. Moreover, TGF-β1 which is proven to initiate activation of the NLRP3 inflammasome and induce epithelial-to-mesenchymal transition with subsequent fibrosis is effectively inhibited by fraxetin administration [41].

The interplay between autophagy and the apoptotic pathways was reported to play a fundamental role in the pathogenesis of doxorubicin-induced myocardial toxicity and serves as an important platform for any treatment strategy for this condition [42]. In the present study, administration of doxorubicin significantly abrogated the mediators of autophagy concomitantly with enhancement of the apoptotic pathways in the myocardial tissues. This was in agreement with El-Agamy et al. [27] who stated that doxorubicin administration was associated with upregulation of mTOR/AKT signaling with subsequent inhibition of MAP kinase and JNK activity in cardiac tissues. These changes induced by doxorubicin were proven to be associated with augmentation of caspase-3 and caspase-9 activity with enhancement of apoptosis [43]. In addition, it was reported that doxorubicin may reduce the tissue levels of the antiapoptotic proteins such as Bcl2 and NGF-β, and thereby lead to overt cytotoxicity [44,45].

In the present study, fraxetin administration was associated with dose-dependent enhancement of the molecular events related to autophagy including downregulation of mTOR/AKT signaling together with enhancement of MAP kinase activity and JNK activity and increased beclin-1 levels compared to rats treated with doxorubicin alone. This was in accordance with the results of Sánchez-Reus et al. [46], who established a strong link between the potent antioxidant and anti-inflammatory properties of fraxetin on one hand and its ability to enhance autophagy in various body tissues on the other hand. Xu et al. [47] reported that fraxetin directly affects mTOR production with subsequent release of MAP kinase from the inhibitory effects created by AKT/mTOR signaling pathway. In addition, Sumorek-Wiadro et al. [48] found that beclin-1, which is enhanced by administration of coumarins including fraxetin, plays a fundamental role in regulation of autophagy/apoptosis balance with the net result of inhibition of the apoptotic events in cardiac tissues, which was in the same line with the data obtained from the present work.

## 5. Conclusions

**Figure 7:**
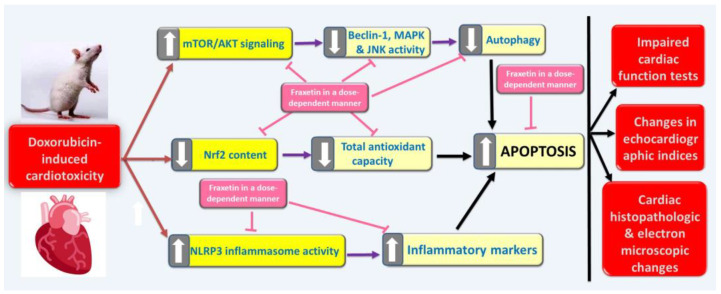
A diagram showing the mechanisms by which the different doses of fraxetin might combat doxorubicin-induced cardiotoxicity.

Fraxetin may be considered as a new adjuvant line of therapy on the way to abrogate doxorubicin-induced cardiotoxicity. This might be due to its effects on ROS production with subsequent affection of NLRP3 inflammasome activity, which is the center point of the pathogenic events that occur in the myocardium of doxorubicin-treated animals. In addition, modulation of autophagy/apoptosis balance may be another mechanism by which fraxetin might restore cardiac functions (Figure 7). Further work is vitally needed to delineate the exact mechanisms that may underlie these desirable effects and to plan future clinical applications.

## Figures and Tables

**Table 1:** Effect of different doses of fraxetin on cardiac function tests in doxorubicin-treated rats (Mean ± SD).

Parameters	Control	Doxorubicin	Doxorubicin + Fraxetin Small Dose	Doxorubicin + Fraxetin Large Dose
Serum CK-MB (U/L)	75.21 ± 8.13	169.6 ± 18.23 ^a^	130.15 ± 14.38 ^b^	105.02 ± 11.7 ^bc^
Serum LDH (U/L)	742.3 ± 80.6	1994.6 ± 180.9 ^a^	1412.8 ± 148.5 ^b^	968.8±101.2 ^bc^
Serum troponin I (ng/mL)	1.73 ± 0.18	3.87 ± 0.41 ^a^	2.72±0.32 ^b^	2.18 ±0.24 ^bc^
Serum NT-pro BNP (ng/L)	8.68 ± 1.06	18.23 ± 1.95 ^a^	13.92 ± 1.53 ^b^	10.27 ± 1.21 ^bc^

^a^ Significant relative to the control group (*p*-value less than 0.05); ^b^ Significant relative to doxorubicin group (*p*-value less than 0.05); ^c^ Significant relative to doxorubicin + fraxetin small dose group (*p*-value less than 0.05).

**Table 2:** Effect of different doses of fraxetin on cardiac tissue p-mTOR, p-AKT, p38 MAP kinase, JNK activity, and beclin-1 levels in doxorubicin-treated animals (mean ± SD).

Parameters	Control	Doxorubicin	Doxorubicin + Fraxetin Small Dose	Doxorubicin + Fraxetin Large Dose
Tissue p-mTOR (% change from the control)	100.00 ± 10.95	191.72 ± 20.22 ^a^	161.1 ± 18.34 ^b^	130.84 ± 15.18 ^bc^
Tissue p-AKT (% change from the control)	100.00 ± 9.89	183.49 ± 19.8 ^a^	155.95 ± 17.47 ^b^	124.17 ± 14.4 ^bc^
Tissue p38 MAPK (% change from the control)	100.00 ± 11.45	56.17 ± 6.91 ^a^	73.84 ± 8.25 ^b^	86.38 ± 9.16 ^bc^
Tissue JNK activity (% change of control)	100.0 ± 12.3	54.3 ± 5.8 ^a^	70.2 ± 7.32 ^b^	81.52 ± 8.82 ^bc^
Tissue beclin-1 (ng/g protein)	8.36 ± 0.94	3.89 ± 0.45 ^a^	5.75 ± 0.61 ^b^	6.97 ± 0.82 ^bc^

^a^ Significant relative to the control group (*p*-value less than 0.05); ^b^ Significant relative to doxorubicin group (*p*-value less than 0.05); ^c^ Significant relative to doxorubicin + fraxetin small dose group (*p*-value less than 0.05).

**Table 3:** Effect of different doses of fraxetin on the echocardiographic indices in doxorubicin-treated rats (Mean ± SD).

Parameters	Control	Doxorubicin	Doxorubicin + Fraxetin Small Dose	Doxorubicin + Fraxetin Large Dose
Heart Rate (bpm)	229 ± 24	225 ± 23	234 ± 26	228 ± 24
LVEDD (cm)	0.59 ± 0.07	0.57±0.06	0.56 ± 0.06	0.61 ± 0.07
LVESD (cm)	0.32 ± 0.04	0.45 ± 0.05 ^a^	0.38 ± 0.04 ^b^	0.34 ± 0.04 ^bc^
LVEF (%)	78.4 ± 8.92	39.4 ± 4.21 ^a^	55.4 ± 5.81 ^b^	68.9 ± 7.12 ^bc^
LVFS (%)	50.3 ± 6.1	24.76 ± 2.6 ^a^	34.5 ± 3.6 ^b^	41.15 ± 4.41 ^bc^
MPI	0.38 ± 0.04	0.21 ± 0.03 ^a^	0.27 ± 0.03 ^b^	0.34 ± 0.04 ^bc^

^a^ Significant relative to the control group (*p*-value less than 0.05); ^b^ Significant relative to doxorubicin group (*p*-value less than 0.05); ^c^ Significant relative to doxorubicin + fraxetin small dose group (*p*-value less than 0.05). LVEDD-left ventricle end-diastolic diameter; LVESD-left ventricle end-systolic diameter; LVEF-left ventricular ejection fraction; LVFS-left ventricular fractional shortening; MPI-myocardial performance index.

## Data Availability

Data is contained within the article.
